# Conjugated Oligo- and Polymers for Bacterial Sensing

**DOI:** 10.3389/fchem.2019.00265

**Published:** 2019-04-18

**Authors:** Susanne Löffler, Haris Antypas, Ferdinand X. Choong, K. Peter R. Nilsson, Agneta Richter-Dahlfors

**Affiliations:** ^1^Department of Neuroscience, Swedish Medical Nanoscience Center, Karolinska Institutet, Stockholm, Sweden; ^2^Department of Chemistry, IFM, Linköping University, Linköping, Sweden

**Keywords:** conjugated polymers, conjugated oligoelectrolytes, luminescent conjugated oligothiophenes, bacterial sensing, biofilms, curli, cellulose

## Abstract

Fast and accurate detection of bacteria and differentiation between pathogenic and commensal colonization are important keys in preventing the emergence and spread of bacterial resistance toward antibiotics. As bacteria undergo major lifestyle changes during colonization, bacterial sensing needs to be achieved on different levels. In this review, we describe how conjugated oligo- and polymers are used to detect bacterial colonization. We summarize how oligothiophene derivatives have been tailor-made for detection of biopolymers produced by a wide range of bacteria upon entering the biofilm lifestyle. We further describe how these findings are translated into diagnostic approaches for biofilm-related infections. Collectively, this provides an overview on how synthetic biorecognition elements can be used to produce fast and easy diagnostic tools and new methods for infection control.

## Introduction

Detection of bacterial pathogens in clinical settings is often based on slow and outdated technology (O'Neill, 2016). Bacterial culturing on solid nutrient media in Petri dishes has remained the golden standard for detection since the 1880s despite lengthy incubation times. Light microscopy and Gram staining are routinely used methods but their specificity and sensitivity are low (Bursle and Robson, 2016). With bacterial infections re-emerging as a global health threat, there is an urgent need to implement new technologies with rapid delivery of results (O'Neill, 2016). Currently, the long diagnostic turnaround times often leave the clinician with no choice but to empirically prescribe antibiotics to secure a patient's life. With no information at hand on the presence and identity of bacteria, this approach may result in suboptimal therapy or unnecessary prescription of antibiotics that may negatively affect the patient's bacterial microflora and select for resistant bacteria (Cantón and Morosini, 2011). New technologies with reduced diagnostic turnaround times should also account for the high complexity of bacterial pathogenesis. Contrary to the planktonic, single-cell lifestyle, bacteria can form multicellular colonies called biofilms. Characteristic of the biofilm lifestyle is that bacteria embed themselves in a self-secreted extracellular matrix (ECM). This matrix, which consists of proteins, extracellular DNA, and polysaccharides, provides spatial organization, and mechanical support to the community (Flemming and Wingender, 2010). The aggregation of proteins and polysaccharides acts as a molecular glue that adheres the bacterial cells to each other and to both biotic and abiotic surfaces. Biofilms thus become extremely resilient toward mechanical or chemical cleaning as well as antibiotic treatment (Hall-Stoodley et al., 2004). Despite evidence for the importance of biofilms for bacterial virulence and the establishment of chronic infections, which are difficult to treat, no diagnostic methods exist to identify a biofilm infection (Phillips and Schultz, 2012; Koo et al., 2017).

In an effort to achieve rapid detection of pathogens, several new approaches have been implemented. These include amplification or hybridization of pathogen-associated nucleic acids, antibody-based immunological detection of bacterial antigens, and various forms of biosensors (Law et al., 2014). While nucleic acid-based and immunological detection methods are highly specific and can detect as few as 10 colony-forming units (CFU/ml), sample processing can be time-consuming, require experienced personnel as well as expensive equipment (Min and Baeumner, 2002; Mollasalehi and Yazdanparast, 2013; Huang et al., 2014). Biosensor-based approaches have a higher detection limit (≥10^3^ CFU/ml) but deliver the result within an hour (Wei et al., 2007). Biosensors for bacterial detection are generally based on an optical or electronic transducer combined with a biological recognition element, such as receptors, nucleic acids, or antibodies (Ivnitski et al., 1999).

Conjugated polymers (CPs), conjugated polyelectrolytes (CPEs), and soluble conjugated oligoelectrolytes (COEs) recently emerged as interesting materials interfacing biological systems. Among important features, these materials show optoelectronic properties, redox activities as well as tunable electronic conductivity (Löffler et al., 2015a,b). CPs and CPEs have been successfully used as actuators and modulators of biological systems and as transducer materials in various biosensors as well as in neuronal interfaces (Gerard et al., 2002; Wang et al., 2002; Svennersten et al., 2009, 2011; Larsson et al., 2013; Simon et al., 2015). This review highlights specifically the use of CPs, CPEs, and COEs for detection of bacteria and bacterial biofilms.

## Bacterial Sensing Using Conjugated Polymer Surfaces

### Detection of Bacteria Based on CP Redox Properties

CPs such as poly(3,4-ethylenedioxythiophene) (PEDOT), polypyrrole (PPY), polydiacetylene (PDA), and polyaniline (PANI), are characterized by a π-conjugated backbone. The π-electron system at the backbone confers optical and semiconducting properties. Electrically conducting polymers can be created by charge injection to the conjugated backbone (doping) and by fabrication of thin films or other microstructures to allow for electronic coupling between the polymer chains. Anionic dopants (counter anions) are embedded into CP films during electrochemical or chemical oxidation to neutralize the positive charges in the CP backbones (Le et al., 2017; Inal et al., 2018). The most well-known CP counter anions are tosylate (Tos), poly(styrenesulfonate) (PSS), and dodecyl benzene sulfonate (DBS). Several other small and biologically relevant anions have also been used, such as heparin, DNA and dexamethasone (Herland et al., 2011; Gomez-Carretero et al., 2017; Shah et al., 2018).

**Figure 1:**
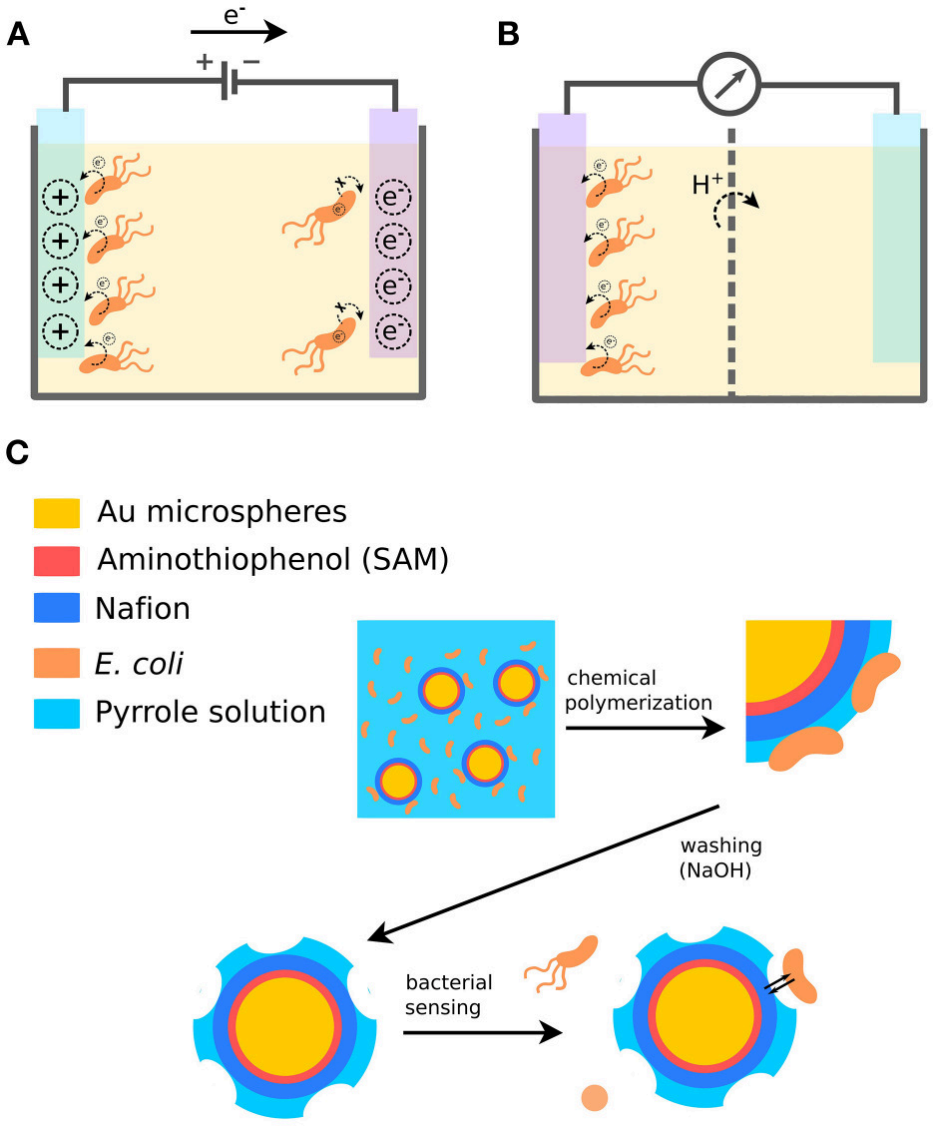
Bacterial sensing using conjugated polymers. **(A)** Modulation of bacterial biofilm formation based on the availability of electron acceptors in the PEDOT surface. **(B)** Detection of bacteria by sensing bacterial electron transfer to a PEDOT electrode. **(C)** Detection of bacteria by fabrication of cell-imprinted microspheres using the conjugated polymer PPy.

Successful modulation of the formation of *Salmonella* biofilm has been demonstrated using electropolymerized PEDOT doped with heparin, DBS, or chloride (Gomez-Carretero et al., 2017). Fabrication of polymer surfaces was performed under conditions that maintained comparable charge storage capacity and hydrophobicity irrespective of counter ion. The electrochemical redox state and exposed chemical functional groups were therefore the sole factors affecting biofilm formation. To investigate whether the redox state of PEDOT, i.e., the amount of available electron acceptors, influences biofilm formation, surfaces integrated into a custom-made culturing device were electronically addressed to generate oxidized, reduced or unswitched (pristine) PEDOT during static culturing of *Salmonella* for 24 h. Macroscopic analysis of the amount of surface-attached biomass revealed a decrease of 52–58% in the reduced state and 39–58% in the unswitched state compared to the oxidized state (Figure 1A). The same effect was observed irrespective of counter ion. Moreover, bacteria themselves were shown capable to reduce the PEDOT (Figure 1B). This work is the first demonstration of a bidirectional mode of redox control, where an intimate interplay between bacteria and the electroactive material defines the bacterial physiology based on the availability of electron acceptors in the PEDOT.

In a similar study, the electrochromic properties of a graphene-PEDOT composite were used to observe electron transfer from *Pseudomonas aeruginosa* (*P. aeruginosa*) to the material (Webb et al., 2015). High conductivity graphene-PEDOT composites are ideal to observe electron transfer occurring between bacteria and substrate, owing to their robust mechanical and enhanced electrocatalytic properties. The color of graphene-PEDOT composite films exposed for 2 weeks to *P. aeruginosa* appeared darker and showed a significant increase in sheet resistance compared to control surfaces. The reduction of the graphene–PEDOT was likely due to the exposure to bacteria. A simple sensor for detection of bacteria on or near the substrate was proposed based on the monitoring of electrical resistance and/or optical absorbance of the graphene–PEDOT.

The mechanism of PEDOT redox control was used for creating a high-throughput, rapid, and highly sensitive test array to evaluate the electrogenic properties of newly discovered and/or genetically engineered bacterial species (Gao et al., 2017). By applying a recent method for co-fabrication of electrofluidic structures on paper (Hamedi et al., 2016), Gao et al. integrated a three-dimensional microbial fuel cell (MFC) device within paper. PEDOT:PSS conductive ink patterned on paper was used as anode reservoir, which was separated from a metal cathode by a wax membrane that allowed for ion exchange. An 8-channel MFC array was fabricated to assay the electrogenicity of wild type *P. aeruginosa* and isogenic strains with mutations in metabolic and signaling pathways as well as in surface structures expected to affect the electrochemical activity of the bacterial cells. Generally, minor differences were detected between the wild type and mutant strains. A hyperpiliated *pilT* mutant, known to show increased cell-to-cell and cell-to-surface adhesion, exhibited increased electrogenicity (Chiang and Burrows, 2003). In contrast, a deletion in the *pmpR* gene, known to inhibit the type III secretion system, generated less current than the wild type (Liang et al., 2012). Collectively, this work presents an important milestone toward redox-based sensing of bacteria using conducting polymers.

### Detection of Bacteria by Cellular Imprinting in Conjugated Polymer Surface

Introduction of recognition sites to the transducer matrix in a biosensor allows for sensitive and specific sensor operation. The most relevant biological recognition elements are protein and DNA receptors. Physical recognition by size and shape is based on the creation of an analyte-shaped mold in the transduction matrix, a technique referred to as molecular imprinting. The technique allows rapid formation of cavities forming receptors for various targets (Haupt et al., 2012). A variety of molecular imprinting applications using conducting polymers have been presented (Hayden et al., 2006; Schillinger et al., 2012; Canfarotta et al., 2018; Parlak et al., 2018).

One example demonstrates a bacterial biosensing application, where a cell-imprinting technique has been used to generate specific binding sites for bacteria, thereby enabling their detection and differentiation (Figure 1C) (Shan et al., 2017). This involves the detection of *Escherichia coli* (*E.coli*) strain O157:H7 in aqueous medium. Cell-imprinted microspheres were fabricated based on a gold-coated plastic microbead core (5 nm) and a polymer complex shell. The beads were pre-treated by depositing a self-assembled aminothiophenol monolayer and coating with Nafion through electrostatic interactions. Finally, in the presence of bacterial cells, Polypyrrole (PPy) was deposited on the pre-treated surface by chemical oxidation. This generated microspheres with bacterial cells entrapped in a conjugated polymer matrix. To remove the bacterial cells and expose bacilli-like surfaces complementary to *E. coli*, the microspheres were treated with 0.1 M NaOH, which also overoxidized PPy through curing and de-doping. When *E. coli* O157:H7 subsequently were incubated with the microspheres, bacterial cells were found specifically captured in the complementary cavities. When other strains were tested, such as *E. coli* O157:HNM, *E. coli* O26:H11, *E. coli* O26:HNM, *E. coli* O rough as well as *P. aeruginosa, Serratia marcescens*, and *Acinetobacter calcoaceticus*, the cross-reactivity of the cell-imprinted microspheres was lower than 10%. The specificity of the imprinted material likely arises from multiple interactions with chemical groups in the cavity as well as from shape and size complementarity to the imprinted bacterial cell. The same basic technique was later used to fabricate cell-imprinted microplates used as biorecognition elements in an ELISA detection assay (Shan et al., 2018). The assay showed high selectivity and sensitivity for the imprinted bacterial cells, demonstrating that CPs as cell-imprinted matrices can complement or even replace biological receptors as superior recognition elements in biosensor applications.

## Bacterial Sensing Using CP Vesicles

**Figure 2:**
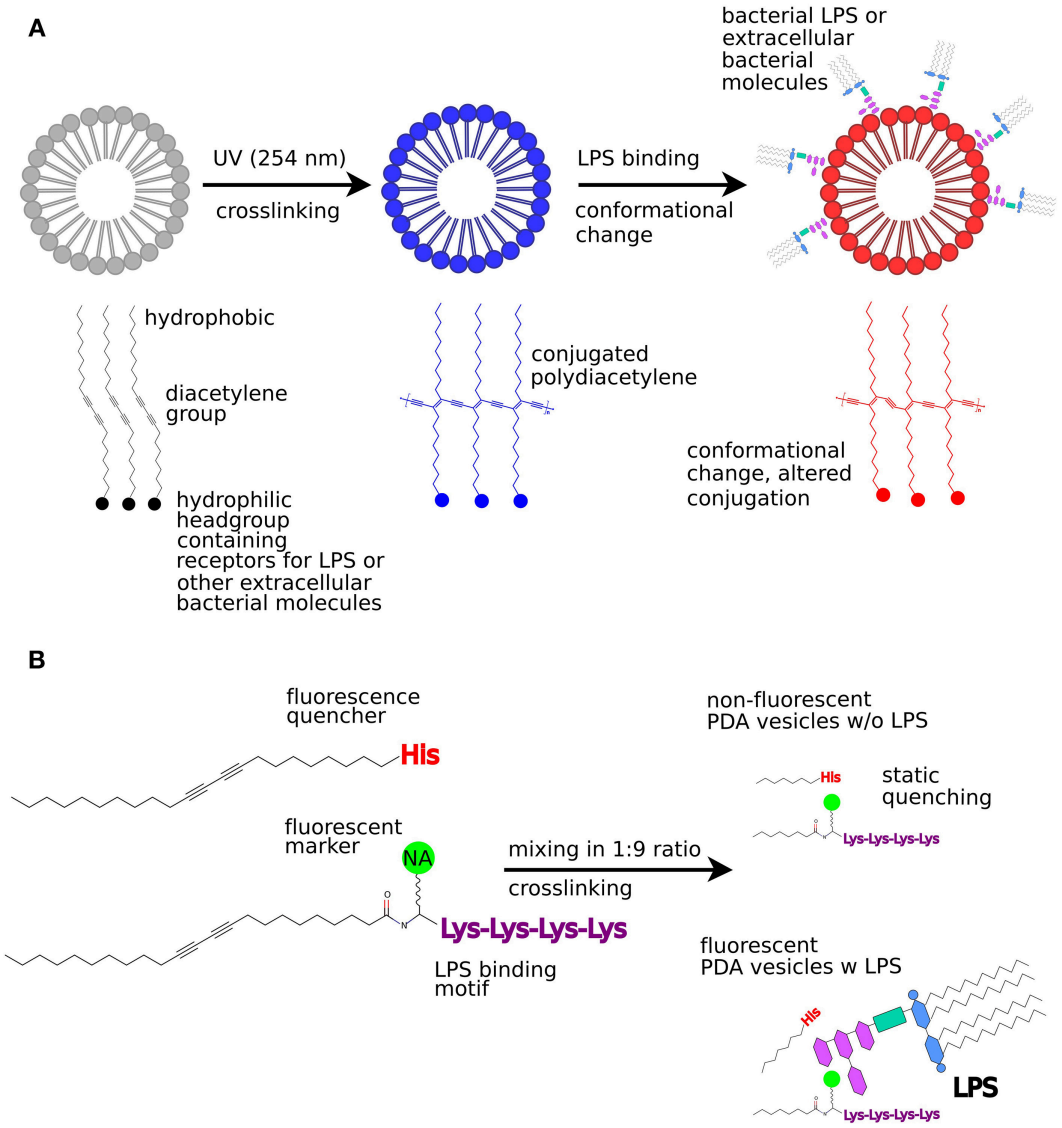
Bacterial sensing using PDA vesicles. **(A)** Colorimetric sensing utilizing absorption change due to conformational change in PDA structures when bacterial LPS binds to the PDA vesicle. **(B)** Fluorescence turn-on sensor for bacterial LPS. This figure was partly reproduced from Kim et al. (2012), licenced under CC BY 3.0 (http://creativecommons.org/licenses/by/3.0/).

The CP polydiacetylene (PDA) has a lipid-like amphiphilic structure. The hydrophobic tail and hydrophilic head group drive self-assembly into sheets or vesicles in aqueous solution (Figure 2A). When aligned, adjacent molecules can be crosslinked using UV irradiation (254 nm), thereby generating a robust polymerized structure. The polymerized, conjugated framework shows increased absorption at 650 nm, appearing as deep blue color. Perturbations of the conjugated framework and conformational changes of the polymeric backbone cause a typical blue to red color transition. Environmental stimuli such as pH, temperature, and pressure, are known to induce such perturbations as well as analytes binding to recognition elements embedded in the vesicle structure (Kaganove et al., 2005; Kim et al., 2012). As the colorimetric transition initiated by analyte binding is visible with the naked eye, rapid sensing platforms can be created based on the PDA liposomes.

The PDA liposome system has been used to detect and differentiate *E. coli* O26:B6, *P. aeruginosa, Salmonella Enteritidis*, and *Salmonella Minessota* based on the specific lipopolysaccharide (LPS) expressed by each strain (Rangin and Basu, 2004). LPS are complex glycolipids anchored within the outer membrane of gram-negative bacteria by the lipidated disaccharide Lipid A. Attached to Lipid A is a core oligosaccharide fragment, which extends the serotype-specific polysaccharide called O-antigen. When carbohydrate binding proteins make contact with sugars, it frequently occurs via tryptophan and tyrosine residues (Weis and Drickamer, 1996). These residues were therefore added as anchors on the PDA liposomes through functionalization of the hydrophilic headgroup. The functionalized PDA liposomes were then self-assembled and crosslinked. Upon exposure of the functionalized PDA liposomes to bacterial LPS, a visible color change from blue to red was observed, consistent with an increase in absorbance at 550 nm. Using a similar approach, PDA liposomes were functionalized with dioctadecyl glyceryl ether-β-glucosides (DGG) as anchor molecules, which enabled detection of *E. coli* observed by a change in peak absorbance from 650 nm (blue) to 550 nm (red) (Ma et al., 1998).

In a different approach, PDA liposomes were used for bacterial detection by means of their secreted compounds. Surfactin is a cyclic lipopeptide that is secreted from gram-positive spore-forming *Bacillus subtilis* (*B. subtilis*) as a natural antibiotic. An amine-functionalized PDA liposome was generated and added to the Luria Bertani (LB) growth medium in agar plates. When comparing the growth of a *B. subtilis* surfactin-producing strain (NCIB3610) to a non-producing strain (SSB466) on the PDA-LB-agar, different phenotypes were clearly distinguished as the color changed from blue to red, in correspondence to an absorbance shift from 645 to 550 nm (Park et al., 2016).

Interestingly, peptide-functionalized PDA liposomes can also act as a fluorescent turn-on sensor for bacterial LPS (Wu et al., 2011). In this approach, the naturally occurring LPS-binding antibiotic polymyxin B (PMB) was used as a template to synthesize an LPS-binding anchor molecule. To mimic the LPS-binding region of PMB, an amphiphilic PDA lipid was synthesized that carried a pentalysine oligopeptide as headgroup, with a naphthalic acid fluorophore linked to the N-terminal lysine. On another amphiphilic PDA lipid, a single histidine residue was added as headgroup. Both amphiphiles were mixed, self-assembled into liposomes and crosslinked so that the histidine group of one component quenched the naphthalic acid fluorescence of the other (Figure 2B). When LPS was added to these mixed PDA liposomes, increased fluorescence from the naphthalic acid was observed. This is most likely because the close contact between the fluorescent unit and the quencher was disrupted when the LPS interacted with exposed pentalysine residues. Despite some cross-reactivity with bovine serum albumin (BSA), these PDA liposomes were successfully used to detect *E. coli* strain DH5α as spectrophotometric recordings as well as confocal microscopy revealed an on-switch of fluorescence from the naphthalic acid.

## Soluble Conjugated Poly- and Oligoelectrolytes for Bacterial Sensing

Characteristic for CPEs are the π-conjugated backbone and ionic pendant groups, which enable electrostatic interaction with oppositely charged counterions and effectively create a conjugated polyelectrolyte. The π-conjugated backbone enables excitation of electrons from the highest occupied molecule orbital (HOMO) in the π-band to the lowest unoccupied molecule orbital (LUMO) in the π^*^ band. Energy in the range of the UV-vis spectrum is required for π-π^*^ transitions. After excitation, electrons relax via rapid emission as photons give rise to fluorescence, or non-radiative decay via heat or energy transfer to acceptors. Thus, conjugated systems have interesting optical properties that can be interpreted using UV-vis spectroscopy. The ionic side chains confer water solubility and the charged pendant groups allow formation of electrostatic interactions with oppositely charged macromolecules (Mcquade et al., 2000).

The number of repeating units in the backbone of CPEs is generally not well-defined. In contrast, conjugated oligoelectrolytes (COEs) contain chemically defined structures as well as a defined number of repeat units. This makes COEs more versatile, reliable, and selective compared to their polymeric counterparts. The conjugated backbone and hydrophilic-charged side chains can easily be modified to tune the optoelectronic properties. Batch variability is minimized due to the defined structure and the limited number of repeat units creates weaker interactions between the COEs and their targets, thereby increasing specificity (Bunz, 2000; Liu and Bazan, 2004).

### Detection of Bacteria Based on Fluorescence Quenching in COE Complexes

Fluorescence quenching can lead to formation of a non-fluorescent complex between the COE and a quencher molecule (static quenching) or to exchange of resonance energy between a donor and acceptor molecule (dynamic quenching). The most common dynamic quenching mechanism is Förster Resonance Energy Transfer (FRET) in which the donor fluorophore transmits energy to the acceptor fluorophore without coming into direct contact. This process is extremely dependent on the donor-acceptor distance, which can be used to detect changes in the distance between them. This allows FRET to be used for detection of the presence of an analyte based on its interaction with a COE complex.

**Figure 3:**
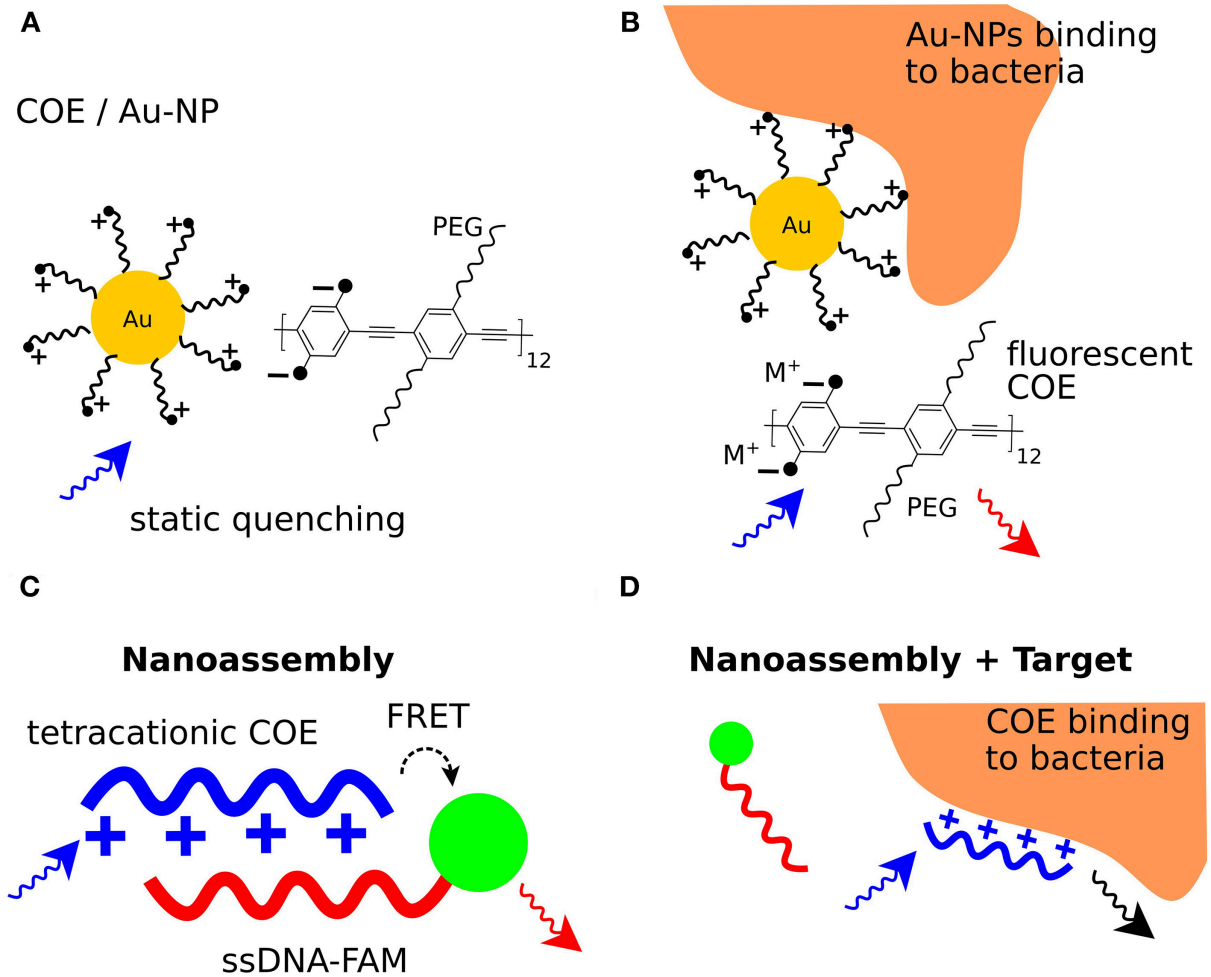
Bacterial sensing using conjugated poly- and oligoelectrolytes. **(A)** Formation of a non-fluorescent complex between ammonium functionalized Au-NPs and the anionic COE (COE/Au-NP). **(B)** Binding of bacteria to the ammonium functionalized Au-NPs frees the fluorescent COE and leads to fluorescence emission at 463 nm. **(C)** Resonance energy transfers from a tetracationic COE donor to the FAM acceptor in a COE/ssDNA-FAM complex. Excitation of the COE leads to FAM fluorescence emission. **(D)** Interaction of bacteria with the tetracationic COE frees the ssDNA-FAM so that excitation of the COE mainly leads to COE fluorescence emission.

A bacterial detection system based on static quenching was demonstrated using cationic Au nanoparticles (NPs) and the anionic conjugated polymer poly(para-phenyleneethynylene) (PPE) featuring a branched oligo(ethyleneglycol) side chain (Phillips et al., 2008). Free PPE is excited at 400 nm and exhibits maximum emission at 463 nm. When the anionic PPE is complexed with cationic ammonium-functionalized Au-NPs, PPE fluorescence is quenched by the formation of supramolecular complexes (Figure 3A). Three different Au-NPs were used exhibiting various hydrophobic anchors close to the ammonium moiety. When the PPE/Au-NP complexes were exposed to bacteria, PPE fluorescence was recovered, indicating that functionalized Au-NPs interact with the bacterial surface (Figure 3B). Exposure of the PPE/Au-NP complexes to a total of 12 different gram-positive, gram-negative, and spore-forming bacteria induced distinct fluorescence changes for each bacterial strain. This indicated that differentiation in the fluorophore displacement could be applied for bacterial identification. Analyzing the fluorescence recovery for each of the three PPE/Au-NP complexes with each bacterial strain, a unique interaction pattern was established. As a dedicated machine-learning algorithm was fed with learning data from all interaction patterns, bacterial identification with an accuracy of >95% was achieved in a second, double-blind experiment using unknown bacterial samples.

A method for bacterial detection based on dynamic quenching was presented using a tetracationic COE based on a fluorene phenylene fluorene (FPF) framework with hexylammonium side chains. FPF was complexed with single-stranded DNA (ssDNA) labeled with fluorescein (FAM). The FPF/ssDNA-FAM complexes were used to detect and differentiate between bacterial strains of *E. coli* K12, *E. coli* FAD-1, *Lactobacillus acidophilus, Rhodopseudomonas palustris* CGA009, *Sporomusa* DMG58, and *Streptococcus mutans* (Duarte et al., 2010). This bacterial sensor is based on resonance energy transfer, where FPF acts as a resonance energy donor and ssDNA-FAM as acceptor. Five different ssDNA sequences containing 20 nucleotides each were labeled with FAM at the 5' end. Mixing each ssDNA-FAM with FPF in phosphate buffer generated five different FPF/ssDNA-FAM composites. When the FPF/ssDNA-FAM composites were excited at 336 nm (Ex_max_ FPF), the optical spectrum showed a small peak at 365–480 nm characteristic for FPF emission, and a larger peak at 490–600 nm characteristic for FAM emission. The presence of these peaks indicated resonance energy transfer from the FPF donor to the FAM acceptor (Figure 3C). Upon mixing with a diluted bacterial culture, the intensity of the FPF peak increased, whereas the intensity of the FAM peak decreased. This suggested an increase in the average distance between the FRET donor-acceptor pair when bacteria interact with each FPF/ssDNA-FAM complex (Figure 3D). Next, diluted cultures of each bacterial strain were mixed with the five different FPF/ssDNA-FAM complexes, and emission spectra were recorded. Comparison of optical spectra from FPF/ssDNA-FAM in the presence vs. absence of bacteria generated a parameter δ, derived from the increase in FPF fluorescence and decrease in FAM fluorescence. As δ differed slightly for each of the five different FPF/ssDNA-FAM complexes, a consistent and distinct pattern was established for each complex interacting with each bacterial strain. To classify the interaction patterns, a discriminant analysis workflow was used, which provided a basis for bacterial identification based on FPF/ssDNA-FAM complexes. In a follow-up study using advanced data modeling (Duarte et al., 2012), the feasibility of this method was extended to discriminate not only between different bacterial strains, but also between bacteria cultured under different conditions. This highly sensitive technique for bacterial differentiation represents an innovative method, equally applicable to microbiologists in the basic research, and clinical laboratories alike.

### Optotracing Based on the Intrinsic Optical Characteristics of COEs for Bacterial Detection

The optoelectronic properties of COEs have shown to be excellently suited as a sensing mechanism for large analytes. The multivalent interactions that govern binding of a COE, via its conjugated backbone and/or the pendant side chains, to a range of biomacromolecules lead to a conformational change in the COEs. Since the conformation is inherently linked to the molecules optoelectronic properties, alteration of the intensity and/or wavelength of the absorbed/emitted light is induced. This conformation-dependent optical detection principle has been vividly explored for optical detection of biomacromolecules (Herrmann et al., 2015; Shirani et al., 2015; Nyström et al., 2017).

**Figure 4:**
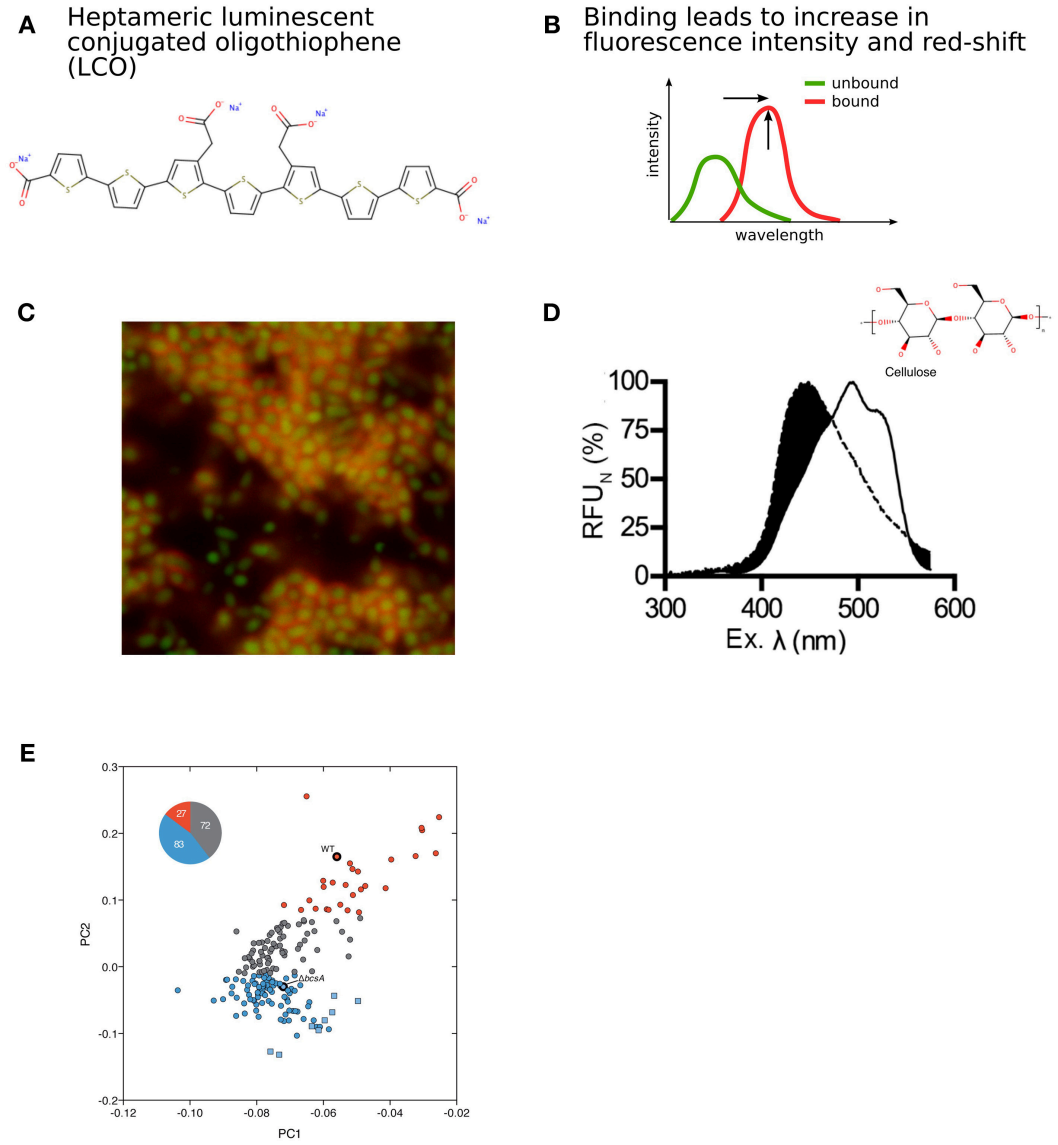
Detection of bacterial biofilm based on modulation of intrinsic optical characteristics of LCOs. **(A)** Structure of a heptameric LCO (h-FTAA). **(B)** Changes in fluorescence intensity and red-shift of the spectrum depending on LCO backbone conformation. **(C)** Confocal imaging revealing large communities of distinct rod-shaped GFP-expressing bacteria (green) surrounded by bacterial biofilm marked by h-FTAA (red). **(D)** Characteristic optical signature of h-FTAA bound to cellulose. **(E)** PCA and k-means clustering of optical spectra from UTI and healthy urine samples screened with LCOs. This analysis identified 27 cellulose-positive (red circles) and 83 cellulose-negative (blue circles) urine samples from UTI patients, as well as 72 UTI urine samples with insufficient discriminatory performance (gray circles). Healthy urine samples (blue squares) were also differentiated from infected samples. Cellulose-positive (WT) and cellulose-negative (△bcsA) biofilm controls are also indicated. Panel **(C)** was reproduced from Choong et al. (2016a), Panel **(D)** was reproduced from Choong et al. (2018) and Panel **(E)** was reproduced from Antypas et al. (2018), licensed under CC BY 4.0 (http://creativecommons.org/licenses/by/4.0/).

Luminescent conjugated oligothiophenes (LCO) represent a class of COEs. Their superior capability for detection of protein aggregates has been utilized for early detection of amyloids in degenerative diseases (Aslund et al., 2009; Klingstedt et al., 2011, 2013; Shirani et al., 2015) (Figure 4A). Upon binding to a target molecule, the flexible conjugated thiophene backbone distorts in response to non-covalent electrostatic interactions. This generates a conformation-based, target-specific spectral signature, which in contrast to conventional fluorophores exhibits ON/OFF fluorescence. Interactions with amyloid proteins characteristically lead to the flattening of the molecular backbone and a more effective conjugation. These changes can be observed as a red-shift in the fluorescence excitation as well as increased fluorescence emission intensity (Figure 4B).

Amyloid proteins are not only relevant in degenerative diseases, but are also produced by bacteria growing biofilm. Amyloid proteins constitute a major component of the extracellular matrix (ECM), in which bacteria embed themselves during the biofilm lifestyle. Key constituents of biofilms produced by *E. coli* and *Salmonella* are the amyloid curli protein and structural polysaccharides. The first sensing platform enabling specific reporting of the presence of biofilm was recently developed (Choong et al., 2016a). The method, termed optotracing, takes advantage of the specific optical signatures generated from heptameric LCOs when bound to curli and cellulose. A selection of isogenic strains of *S. Enteritidis* was used, whose curli and cellulose production is genetically well-defined. Bacterial strains included the wild type *Salmonella* 3934 able to produce both curli and cellulose (curli+/cellulose+), and the mutants Δ*csgA* producing cellulose but no curli (curli–/cellulose+), Δ*bcsA* producing curli but no cellulose (curli+/cellulose–), and Δ*csgD* which is unable to produce either component of the biofilm matrix (curli–/cellulose–) and therefore cannot grow biofilm. As a first step, LCOs in aqueous solution were added onto pre-formed *Salmonella* biofilm grown on glass surfaces. Confocal fluorescence microscopy revealed distinct labeling of the biofilm formed by curli and/or cellulose producing strains, thus demonstrating a novel use of the LCOs for visualization of the distinct biofilm morphology.

To widen the applicability of the method to live studies, the toxicity of LCOs was tested. No effects were observed on bacterial growth rates in liquid culture. This corroborates previous studies on eukaryotic cells and intravital mouse models, showing a non-toxic nature of the LCO family of molecules (Aslund et al., 2009). This opened for the use of LCOs as optical sensors enabling kinetic recordings of biofilm production in live bacterial cultures. To test this concept, LCOs were added directly to the culture medium. Spectrophotometric recordings of the growing culture identified one LCO, whose distinct red-shift in excitation clearly differentiated biofilm-forming and non-forming strains based on their production of curli and cellulose. Moreover, a peak appearing at 480 nm could be specifically linked to the cellulose-producing strains. By comparing the signal-structure relationships of the tested LCOs vs. the different bacterial strains, it was found that bi-directional projection of carboxylic side chains along the conjugated thiophene backbone is required for curli and cellulose detection.

Bacterial expression of the curli- and cellulose-encoding genes is under strict regulatory control. When the optotracing method was applied to liquid cultures of wild type *Salmonella*, a progressive increase of fluorescence intensity was observed around 15 h. The increase in fluorescence reflects that at this time point, bacteria have synthesized curli and cellulose, and cells are now in the process of secreting these biomacromolecules into the extracellular environment. When present in the medium, curli and cellulose become available as binding targets for the LCO, which produces biofilm-specific fluorescent signals when bound. Interestingly, the timing coincides with the time point when the bacterial culture transitions from late logarithmic to stationary phase, when a lifestyle-shift from single planktonic cells to the sessile state of biofilm occurs. This was confirmed by confocal imaging. Large communities of distinct rod-shaped GFP-expressing bacteria were observed, surrounded by dense mesh-like structures that were visualized by LCOs bound to curli and cellulose in the ECM (Figure 4C). The optotracing sensing platform thus provides the first method for kinetic recordings of biofilm formation under live conditions.

Sensing of cellulose in the bacterial biofilm was the first report on LCO binding to polysaccharides. In a series of follow-up studies, the molecular details of the interaction between LCO, cellulose and related glucans have been studied (Choong et al., 2016b, 2018). Using the cellulose fragments cellopentaose, celloheptaose, and cellooctaose, a minimum of 8 β(1–4) linked glucose monomers was defined for LCO to bind and give rise to the characteristic cellulose peak at 480 nm (Figure 4D). This interaction provides resolution at the molecular level and discriminates cellulose from highly similar stereoisomers and other glucans. This study opens a new field in which the optotracing technology is used for determination of purity and localization of cellulose in plant and organic biomass of other origins.

The optical sensing platform for bacterial biofilms has also found clinical application, since bacterial biofilm formation is of great clinical significance (Flores-Mireles et al., 2015). Uropathogenic *E. coli* (UPEC) is the main causative agent of urinary tract infections (UTI), where biofilm is considered a main contributor to chronic and recurrent infections. Similar to *Salmonella*, the ECM of UPEC biofilms is mainly composed of cellulose and curli. This inspired Antypas et al. to investigate whether cellulose can be detected in urine by optotracing, as a diagnostic biomarker for UTI (Antypas et al., 2018). Analysis of spectra from LCOs interacting with relevant control strains showed the ability of the LCO to identify cellulose in UPEC biofilms. This was observed as a distinct spectral signature with a primary peak at 464 nm and a secondary at 484 nm. Following successful detection of cellulose in UPEC biofilm *in vitro*, optotracing was applied to urine samples from 182 patients presented with UTI and 8 healthy volunteers. A workflow was developed, where the spectral information from each sample was analyzed by principal component analysis (PCA) and k-means clustering (Figure 4E). This study classified 27 urine samples as positive for cellulose, providing the first, direct evidence that bacteria can form biofilm within the urinary tract and establishing that UTI can be biofilm-related. Interestingly, the method was also able to differentiate healthy from infected urine. Optotracing by LCOs thus represents the first method able to detect biofilm in clinical samples, which may be of high clinical value when selecting for optimal antibiotic treatment.

## Conclusion

This review summarizes recent efforts in which conjugated polymers and conjugated poly-/oligoelectrolytes have been applied for detection and differentiation of bacteria, their secreted compounds as well as the different lifestyles. The variety of applications clearly demonstrates the versatility of these materials to produce simple, sensitive, and fast detection systems to monitor the presence of bacteria, also under clinical conditions. Modulation of bacterial metabolism using the redox state of electroactive surfaces is an unexplored method, which promises great potential for the development of anti-bacterial and anti-biofilm surfaces. Likewise, these materials provide the possibility to read-out bacterial electron transfer processes, which is a field that only recently has been recognized and is likely to gain in importance as extracellular electron transfer mechanisms are described for an increasing number of bacterial species. The specificity of bacterial cell-imprinted matrices made from conjugated polymers highlights how these materials can compete with DNA and antibodies as biological recognition elements, possibly leading to cheaper and more robust biosensing applications in the future. While the tunable optical properties of conjugated polymers can be applied to obtain visible optical information, conjugated oligoelectrolytes often provide extremely sensitive fluorescence readouts. As a wide variety of anchor molecules or counter ions can be used that generate slightly different patterns for each bacterial species, these systems are excellently suited to develop systems like the electronic nose or tongue for bacterial determination. With the use of advanced machine learning techniques, which are more and more easy to use and apply, these systems may also find importance in clinical settings. As the first technology to specifically detect bacterial biofilm and differentiate between different biofilm components, the optotracing technology sets a milestone for detection of sporadic and chronic infections using an optical biosensor approach. Taken together, the availability of all these technologies promises that point-of-care bacterial diagnosis and targeted antibiotic treatment will soon become a clinical reality.

## Author Contributions

All authors listed have made a substantial, direct and intellectual contribution to the work, and approved it for publication.

### Conflict of Interest Statement

All authors are co-inventors of patents (granted and pending) relevant to this work. Intellectual properties are owned by Richter Life Science Development AB founded by AR-D. SL, FC, and KPRN are shareholders of Furcifer AB.
